# Learning research systems: A conceptual framework and formative implementation case

**DOI:** 10.1017/cts.2026.10801

**Published:** 2026-07-21

**Authors:** Laura Lienemann, Carrie Bailey, A. Jerrod Anzalone, Matthew Rizzo

**Affiliations:** 1 Neurological Sciences, University of Nebraska Medical Center, Omaha, NE, USA; 2 Biostatistics, University of Nebraska Medical Center, Omaha, NE, USA

**Keywords:** Learning health system, learning research system, translational science spectrum, implementation science, sociotechnical systems

## Abstract

Learning Health Systems (LHSs) enable data-driven improvement in clinical care. However, research processes that generate this evidence remain fragmented, limiting systematic learning across study portfolios and contributing to persistent translational inefficiencies. We introduce the Learning Research System (LRS) as a sociotechnical extension of the LHS that leverages LHS infrastructure to render research operations measurable, reusable, and governable across studies, positioning research as an object of continuous learning. Using targeted literature synthesis and supplementary searches, we traced the emergence of the LRS from foundational LHS scholarship and developed five core capabilities as proposed maturity indicators: cross-study measurement, reuse of cohorts and analytic assets, portfolio-level governance, feedback-enabled workflow optimization, and integration of research outputs into clinical and operational improvement. We illustrate the distinction between LHS and LRS roles across the NCATS Translational Science Spectrum. Finally, we applied the capabilities to a formative case at the University of Nebraska Medical Center through an institutional environmental scan, mapping them as indicators of emerging LRS maturity from an existing LHS. The LRS provides a framework for evaluating research-process learning, reducing fragmentation across translational stages, and addressing selected translational discontinuities, such as early-stage “valley of death” barriers between discovery and human studies.

## Introduction

Learning Health Systems (LHSs) integrate real-world data, analytics, and feedback loops to improve clinical care continuously [1,2]. Although research is often the engine of this learning cycle, systematic learning about the research process itself remains largely implicit. Within many LHS environments, research projects are managed as discrete projects, with limited cross-study measurement, feedback, or reuse of operational knowledge, resulting in fragmentation that constrains efficiency, generalizability, and scalability across institutional research portfolios.

The LHS incorporates both external and internal evidence and encompasses embedded and pragmatic trials, implementation science, and observational research [3]. Research contributes to clinical improvement within the LHS but is not itself typically the explicit target of systematic process optimization. The concept of the Learning Research System (LRS) emerged from this gap by extending learning system principles to the research lifecycle. We define the LRS as a research-focused sociotechnical extension of the LHS that supports coordinated data management, measurement, analysis, and continuous learning across studies to improve research processes, including study design, cohort identification, recruitment, execution, and data quality. In this sense, the LRS renders core research assets and workflows increasingly findable, accessible, interoperable, and reusable [4].

LHS and LRS infrastructures may coexist or operate independently. A health system can function as an LHS without a major research enterprise by integrating internal and external evidence into care delivery and quality improvement. Conversely, an organization without healthcare delivery can function as an LRS by applying learning-system principles to research workflows across translational stages and study types. For example, a research-intensive university can continuously improve study design, model systems (including animal models), analytic pipelines, and evidence synthesis and dissemination without a healthcare delivery partner. In integrated environments, especially academic medical centers, LHS and LRS activities intersect through reciprocal learning. Routine care generates data that inform hypothesis generation and study design, while research generates new knowledge for dissemination and implementation. Thus, clinical care and research inform each other through a reciprocal learning cycle that strengthens both.

The NIH Data Count Once Use Numerous TimeS (Data COUNTS™) initiative is a national effort to strengthen real-world data infrastructure for research by supporting trusted data brokerage, reusable data resources, privacy-preserving analytics, data-access workflows, and public-private partnerships [5]. UNMC is participating as a pilot partner institution in the initiative toward an NIH Real-World Data Network. Data COUNTS™ is relevant to the LRS framework because it addresses core requirements for learning across studies and sites, including data reuse, multisite governance, standardized data models, secure analytic workflows, and feedback mechanisms that support continuous improvement in research operations.

Drawing on key LHS scholarship, we propose a formal definition of the LRS as conceptually distinct from the LHS while situating both within a broader organizing framework. We define five core capabilities of the LRS as a basis for assessing institutional maturity in LRS implementation and illustrate these capabilities through formative initiatives at the University of Nebraska Medical Center (UNMC), which is developing an LRS aligned with NIH Data COUNTS™ principles [5].

## Methods

### Literature synthesis

We conducted a targeted literature synthesis to identify influential, conceptually relevant works on LHSs, embedded research, research process learning, and emerging LRS-related frameworks. The authors screened titles, abstracts, and full texts for relevance to research-process learning, cross-study measurement, governance, asset reuse, feedback loops, and research-to-practice integration. Project-specific reports that did not substantively inform the LRS construct were excluded. Included works were then organized to trace the conceptual emergence of the LRS and to identify candidate capabilities that could serve as maturity indicators. Disagreements regarding study selection or organization were resolved by consensus.

Themes identified in the full-text review informed a set of candidate capabilities underpinning an LRS. An organization was considered to approach full LRS maturity through cumulative development of the following capabilities: (i) C1: Cross-study measurement. Measure research processes across studies, including timelines, recruitment efficiency, preclinical resource utilization (e.g., animal census), data quality, and analytic reproducibility. (ii) C2: Asset reuse. Reuse analytic and protocol assets, such as cohorts, code libraries, consent templates, and governance pathways, to accelerate subsequent studies. (iii) C3: Portfolio-level governance. Govern the research lifecycle at the portfolio level rather than treating projects as isolated initiatives. (iv) C4: Feedback-enabled learning. Operate feedback loops that link performance data and study findings to the design and execution of new research across translational stages. (v) C5: Research-to-practice integration. Integrate research outputs into clinical and operational improvement to enable continuous learning across the system.

To illustrate how C1–C5 manifest in practice, we conducted an environmental scan of UNMC initiatives by reviewing institutional records, strategic plans, and grant materials, supplemented by consultations with UNMC and Nebraska Medicine faculty and staff representing research leadership, informatics, regulatory affairs, and frontline investigator perspectives. These informal consultations addressed governance, data architecture, analytic environments, clinical-research interfaces, dissemination and implementation processes, and workforce development, informing milestone interpretation and C1–C5 alignment.

## Results

### Emergence of the LRS concept

**Figure 1.**
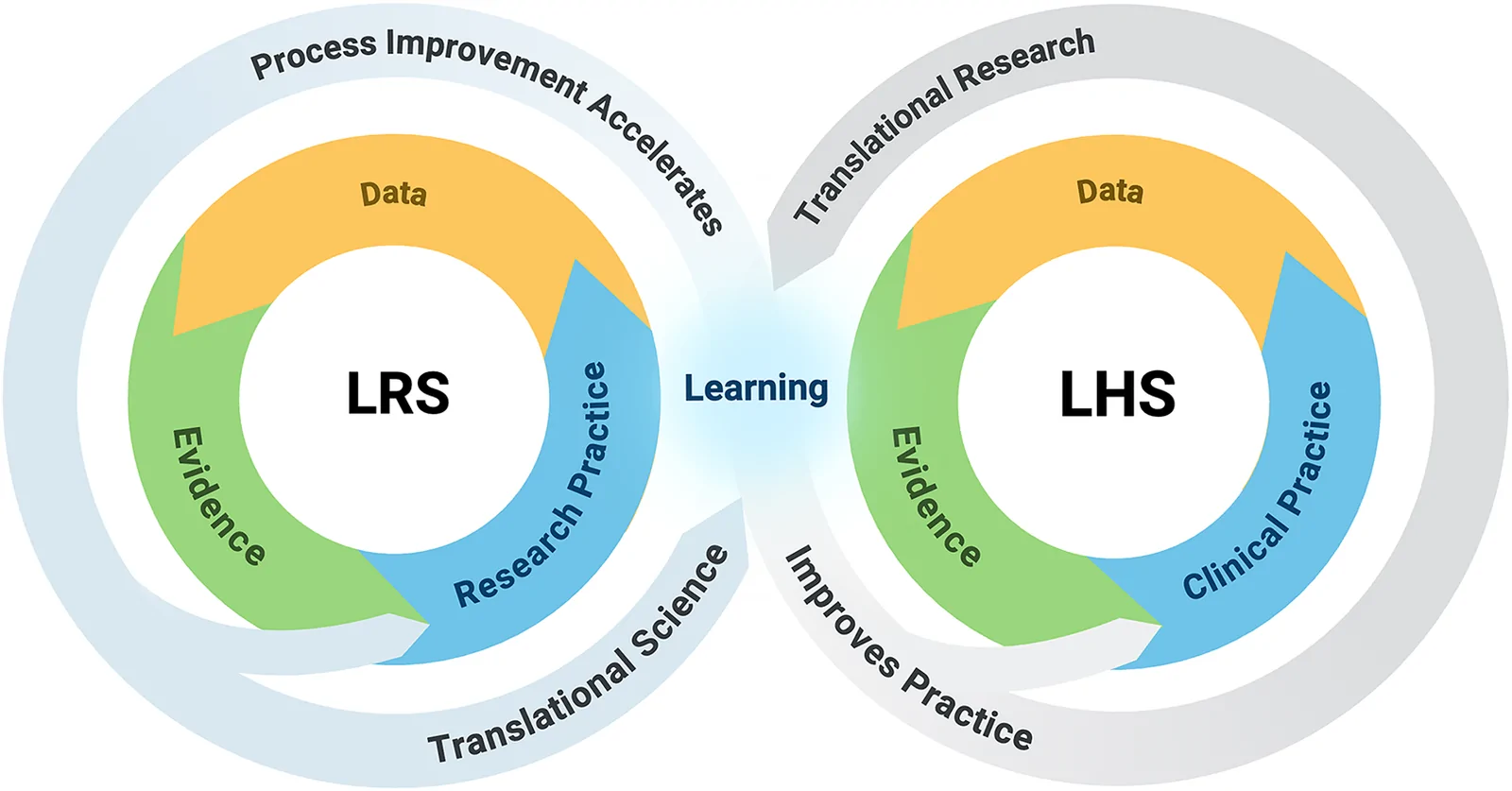
Figure 1 long description. LHS and LRS sociotechnical architectures. The learning research system (LRS) is a research-focused sociotechnical architecture conceptually linked to the learning health system (LHS) through shared learning processes. Whereas the LHS uses research to improve care, the LRS applies learning principles to improve the conduct of research itself, analogous to the distinction between translational science and translational research [7]. Shared governance, data standards, secure analytics, and related capabilities enable bidirectional feedback loops consistent with NIH Data COUNTS™ principles in academic medical centers [5]. Adapted from Agency for Healthcare Research and Quality [3]. Created in BioRender. Lienemann, L. (2026) https://biorender.com/2slvi7g.

Figure 1 depicts the distinct yet overlapping relationship between the LHS and LRS, informed by our literature synthesis, informant consultations, Agency for Healthcare Research and Quality (AHRQ) LHS definitions, and the National Center for Advancing Translational Sciences (NCATS) Translational Science Spectrum [3,6].

Table 1 summarizes seminal literature supporting the emergence of the LRS concept from LHS scholarship. Early LHS literature emphasized rapid-cycle clinical improvement and integrating research with routine care [1,8,9] with limited attention to cross-study process learning. Subsequent scholarship centered on embedded research within LHSs, expanding to practical applications for research process improvement through recruitment monitoring, risk-based oversight, and multi-institutional coordination [2,10,11], but did not establish structures spanning the full translational spectrum. Contemporary scholarship explicitly treats research itself as an object of systematic learning, advancing cross-study measurement, asset reuse, structured feedback, and coordinated regulation consistent with LRS criteria across the translational spectrum [12–15]. Ordering from top to bottom reflects increasing functional integration rather than strict linear stages.

**Table 1.** Seminal literature illustrating conceptual emergence of the LRS. The literature illustrates approximate progression from care-centered learning models (top), to intermediate research-support infrastructures, to enterprise-level, continuous learning (bottom) Table 1 long description.

Citation	Core contribution
Friedman et al., 2010 [1]	LHS linking research to clinical improvement (C5); no cross-study governance (C1–C3)
Greene et al., 2012 [8]	Bidirectional practice–research learning (C4–C5); no portfolio governance (C3)
Psek et al., 2015 [9]	LHS operations transferable to research oversight (C3–C4)
Budrionis et al., 2016 [16]	Learning architectures (C2); limited cross-study measurement (C1)
Friedman et al., 2017 [17]	Cyber-social LHS model enabling research-process learning (C1–C3)
Abernethy et al., 2010 [18]	Embedded oncology research within care; iterative outcome learning (C4–C5)
Guise et al., 2018 [19]	Computable knowledge dissemination; limited research observability (C5)
Forrest et al., 2018 [20]	LHS workforce competencies relevant to successful health systems (C3; C4 contributory)
Platt et al., 2020 [21]	LHS evaluation synthesis (C5); no portfolio research governance (C3)
IOM, 2013 [2]	Regulatory coordination enabling LHS foundation (C3)
Lipman et al., 2014 [22]	Practice networks enabling cohort reuse and governance (C1–C3)
Riley et al., 2013 [23]	Research monitoring/feedback (C1, partial; C4); limited cross-study reuse (C2)
Dizon et al., 2018 [10]	Digital trial tools; partial research-process insight (C1, C3)
Velstra et al., 2020 [11]	Risk-based monitoring enabling cross-study observability (C1)
Denny et al., 2021 [24]	Outlines LHS capabilities that enable precision medicine (C2)
Presley et al., 2025 [7]	Conceptual translational impact learning; emphasizes evaluation over systems operations (C1–C3)
Rosenthal et al., 2023 [15]	Outlines an LHS framework contextualized for academic medical centers based on differentiating features (C4–C5)
Comellas et al., 2023 [12]	Network-level cross-study metrics, reuse, governance (C1–C3)
Goligher et al., 2024 [13]	Adaptive platform trials with automated research learning (C1, C4)
Rizzo, 2025 [14]	Coupled LHS–LRS architecture with AI-enabled feedback (C1–C5)
Huerta et al., 2025 [25]	Presents an LRS in the form of a research-oriented LHS (C1–C5)

### Functional differentiation across the NCATS Translational Science Spectrum (T0–T4)

Table 2 distinguishes LHS and LRS functions across the NCATS Translational Science Spectrum [6]. The LHS uses clinical data to improve care, while the LRS applies learning-system principles to improve research design, activation, execution, monitoring, reuse, and translation. At the population level, LRS functions may include federated analytics, multisite governance, and population-responsive study design.

**Table 2.** LHS and LRS functions across the NCATS Translational Science Spectrum. Across translational stages (T0–T4), the learning health system (LHS) and learning research system (LRS) may operate independently or in coordination, the latter particularly at academic medical centers. The LRS provides infrastructure for cross-study learning, process optimization, and resource reuse Table 2 long description.

Stage	Primary LHS functions	Primary LRS functions
**T0: Basic and Foundational Research**	Identification of unmet clinical needs from real-world evidence and practice.	Governance and regulatory compliance streamlining, protocol and workflow template development, data quality and analyses monitoring, educational needs and resource assessments.
**T1: Preclinical Research**	Signal detection from clinical data; workflow-linked feasibility and pilot studies.	Streamlined cohort identification, testing via animal models, recruitment monitoring, feasibility study metrics, and adaptive protocol support.
**T2: Clinical Research**	Pragmatic trials embedded in care; decision support implementation; real-world validation of interventions.	Trial execution monitoring, harmonization across sites using standard data models, and analytic reproducibility.
**T3: Clinical Implementation**	Continuous learning cycles; comparative effectiveness research; operational optimization and quality improvement.	Post-market evidence generation; rapid linkage of research outputs to operational data; evaluation of adoption and implementation fidelity.
**T4: Public Health**	Population outcomes monitoring; predictive analytics for risk stratification; disparity identification and equitable implementation at scale.	Linkage with public health, environmental, and community datasets; federated (privacy-preserving) analytics; community-engaged research.

Federated analytics may include mapping local data to common standards, running shared analytic code locally at participating sites, and returning aggregate results or model parameters rather than identifiable patient-level data. Multisite governance may include reliance agreements, data-use agreements, common access procedures, shared standard operating procedures, harmonized institutional review board (IRB) and contracting workflows, and cross-site data-access committees. Population-responsive study design may include monitoring recruitment, retention, missingness, protocol deviations, and outcome ascertainment by rurality, geography, race, ethnicity, age, sex, language, disability, insurance status, and other relevant population characteristics, followed by workflow changes when disparities or bottlenecks are identified.

### Formative LRS case study

UNMC is a public, academic health sciences center, closely affiliated with Nebraska Medicine, its primary clinical partner and a 501(c)(3) nonprofit health system. UNMC is located in Nebraska, an NIH Institutional Development Award (IDeA)-eligible state, a designation used by NIGMS for states and jurisdictions that have historically received lower levels of NIH funding and are targeted for biomedical research capacity-building through faculty development, infrastructure enhancement, and clinical and translational research programs.

In fiscal year 2025, UNMC had approximately 1937 faculty, including 523 faculty investigators, annual research expenditures of approximately ＄280 million, and an annual operating budget of approximately ＄1.1 billion. Nebraska Medicine, a ＄3 billion per year academic health system, provides the principal clinical environment for translational and clinical research activities. This context places UNMC among academic health centers with substantial clinical, research, and community engagement infrastructure. It also distinguishes UNMC from larger institutions with multiple nationally designated centers, CTSA hubs, NCI-designated comprehensive cancer centers, or more decentralized research authority.

At UNMC, portfolio-level research governance is coordinated through the Office of the Vice Chancellor for Research (VCR), which reports directly to the Chancellor’s Office. The VCR office works with college deans; center and institute directors; the IRB; sponsored programs; clinical research operations; the Great Plains IDeA-CTR Network; informatics and data-governance resources; and Nebraska Medicine leadership to align research priorities, regulatory workflows, investigator support, data access, and institutional investments. The VCR office does not replace college, departmental, center, or health-system authority. Rather, it coordinates cross-cutting functions that require institutional alignment, including research administration, shared infrastructure, regulatory support, data governance, clinical research services, pilot programs, and translational research strategy.

This governance model should be interpreted as a formative institutional case rather than a universal blueprint. Institutions with multiple major federally funded programs, nationally designated centers, or more decentralized authority may require different mechanisms to manage competing priorities, overlapping data-governance structures, parallel regulatory workflows, and program-specific commitments. For the LRS framework, the relevant feature is not the replication of a specific reporting structure, but the presence of accountable, transparent, and coordinated governance across the research enterprise.

Against this institutional and governance background, Table 3 summarizes selected milestones that contributed to the development of learning research system capabilities at UNMC.

**Table 3.** Selected institutional milestones toward learning research system maturity at UNMC Table 3 long description.

Year	Milestone	Primary LRS capability	Research-system function	Evidence or metric	Contribution to LRS maturity	Example of remaining gap
2004	Vice Chancellor for Research office established	C3	Portfolio-level governance	Supports 6 colleges, 2 institutions, 7 NIH-funded major programs, and 25+ cores/shared resources	Creates an institutional mechanism for cross-cutting research governance	Clarify decision rights and conflict-resolution mechanisms
2008	Center for Collaboration on Research Design and Analysis established	C1, C2	Cross-study measurement and reusable analytic support	Supports approximately 400 projects and 8000 hours annually	Provides shared design and biostatistics infrastructure across studies	Cross-study operational measures remain incompletely standardized
2013	Nebraska Biobank established	C2, C3	Reusable biospecimen and data asset	More than 254,000 samples from approximately 154,000 patients through 2023	Enables reuse of shared biospecimen and linked data assets	Linkage to study performance and implementation feedback remains developing
2014	Research Information Technology Office established	C1, C2	Shared computational, storage, and consulting infrastructure	Supports more than 8000 users	Provides reusable data and computing assets for investigators	Integration with portfolio-level research metrics remains incomplete
2014	UNMC joined the Great Plains Collaborative and PCORnet	C1, C3	Standardized multisite collaboration and data governance	Access to data from more than 47 million patients	Extends common data models and network governance beyond a single institution	Local-to-network feedback loops require further operationalization
2016	GP IDeA-CTR Network initially funded	C3, C4	Regional governance, workforce development, community engagement, and study support	Eight institutions, regional partners, 104 PBRN sites, more than 1200 publications, 153 CEO consultations, and 313 community-engaged research partners	Provides network scaffold for coordinated study activation, monitoring, and community engagement	Portfolio-wide measures of efficiency, equity, and implementation impact remain under development
2016	Biomedical Informatics and Cyberinfrastructure Core launched	C1, C2	EHR data access, cohort identification, and reusable informatics workflows	Data capacity expanded to more than 1.5 million patients; 261 EHR data requests fulfilled since January 2024	Supports cross-study measurement and reusable real-world data workflows	Requires broader linkage to regulatory, recruitment, and implementation metrics
2022	FHIR interoperability extended to 10 critical-access Nebraska hospitals	C1, C2, emerging C5	Interoperable data exchange and rural clinical-research infrastructure	Ten critical-access hospitals connected through FHIR-enabled infrastructure	Establishes groundwork for research-to-practice integration in rural settings	Does not yet constitute mature bidirectional research-practice feedback
2023	READI Core and CRANE expanded	C1, C2, C3	Harmonized EHR extraction, cohort identification, feasibility assessment, and data-quality monitoring	De-identified, HIPAA-compliant warehouse aligned with PCORnet CDM and i2b2 [26]; tracking of cohort yield, request-to-extract time, completeness, quality, and reproducibility	Supports reusable real-world data workflows and portfolio-level monitoring	Requires broader integration with regulatory, recruitment, and implementation workflows
2025	NIH Data COUNTS™ planning and partner-site alignment	C3, C4	Governance and data-access workflow alignment	Planning with nine partner sites for governance and data-access workflows [5]	Aligns local LRS development with emerging national real-world data principles	Operational network metrics and multisite validation remain future work
2025	Nebraska Medicine Application Rationalization Delta Team assembled	C4	Workflow optimization and redundancy reduction	Tasked with reducing redundancy across more than 1200 software applications	Illustrates feedback-enabled enterprise optimization relevant to research workflows	Specific research-process metrics and downstream effects require measurement
2026	Data & Analytics Enablement Program	C1, C2, C3, C4, emerging C5	Modernizing the Nebraska Medicine and UNMC data ecosystem for enhanced data security, governance, and reuse	Establishes contracts to improve computing infrastructure and update systems configuration [27]	Improves research workflows and feedback by increasing data storage and analytic capacity while enhancing security and governance structures	Requires documentation of routinely deployed tools (e.g., risk models, pathway logic, or clinical digital twin benchmarks) with measured impact on clinical outcomes and operational performance, along with feedback loops for ongoing refinement.

C1 = Cross-study measurement; C2 = Asset reuse; C3 = Portfolio governance; C4 = Feedback-enabled learning; C5 = Research-to-practice integration; CEO = Community Engagement and Outreach; CRANE = Clinical Research Analytics Environment; EHR = electronic health record; FHIR = Fast Healthcare Interoperability Resources; GP IDeA-CTR = Great Plains IDeA Clinical and Translational Research; HIPAA = Health Insurance Portability and Accountability Act; i2b2 = Informatics for Integrating Biology and the Bedside; PCORnet = National Patient-Centered Clinical Research Network; PBRN = practice-based research network; READI = Research, Education, Administration, and Development of Biomedical Informatics; UNMC = University of Nebraska Medical Center.

Table 3 is intentionally selective. It is not an inventory of UNMC research assets, but a maturity map of milestones with direct, traceable links to C1–C5. Many academic health centers have IRBs, CTMS platforms, REDCap, data warehouses, biobanks, clinical research units, community engagement programs, and analytic cores. The LRS construct emphasizes whether these assets are connected through shared governance, reusable workflows, interoperable data, cross-study measurement, feedback loops, and research-to-practice integration.

Establishment of the VCR office represents progress toward portfolio-level governance (C3) by formalizing research leadership. This coordinating function has united resources and services for investigators and represented research needs in enterprise-level discussions. UNMC conducts cross-study measurement (C1) through the Center for Collaboration on Research Design and Analysis, the Nebraska Biobank, and the Research, Education, Administration, and Development of Biomedical Informatics (READI) Core, although these measures remain strongest within studies that each resource supports. The IRB tracks all studies submitted for review. GP IDeA-CTR Network tracks studies related to the network and partner sites. The VCR tracks research expenditures, patents, and invention notifications, and the Clinical Research Center provides study intake, services, standard operating procedures, staff education, recruitment, billing, awards, new faculty onboarding, and guidebooks through the Office of Research Integration and Activation. Centers and services hosted by the VCR can be contracted by individual investigators, enabling asset reuse (C2).

These activities demonstrate progress toward feedback-enabled research operations aligned at the enterprise, network, and national levels (C4). Workflow optimization initiatives, including the Nebraska Medicine Application Rationalization Delta Team, reflect system-level efforts to reduce redundancy and improve efficiency. At the regional level, the GP IDeA-CTR Network provides a scaffold for coordinated study activation, governance, and monitoring across sites (C3–C4). In parallel, the READI Core and related informatics resources, including the Clinical Research Analytics Environment (CRANE) enterprise data warehouse for research [26], support harmonized data capture, cohort identification, and alignment with national standards (C1–C2). Strategic planning with NIH Data COUNTS™ partners further aligns governance and data-access workflows with emerging national principles for access to quality data (C3–C4). While establishment of FHIR at 10 critical-access Nebraska hospitals lays groundwork for research-to-practice integration (C5), this effort toward data interoperability does not yet constitute a mature bidirectional system in which research findings routinely feed back into care and practice-generated data routinely improve research design. Taken together, the operational indicators in Table 3 underscore both progress toward an LRS and the need for continued development of C1–C5 capabilities at UNMC.

In the UNMC case, C1 is reflected in shared data resources, feasibility queries, data-quality monitoring, and emerging composite research measures (CRMs); C2 in reusable biobank, informatics, analytic, and clinical research service assets; C3 in VCR-led coordination, GP IDeA-CTR Network governance, data-access workflows, and shared research services; C4 in workflow optimization [27,28], recruitment and feasibility monitoring, and alignment with Data COUNTS™ principles; and C5 as an emerging capability supported by interoperable data infrastructure and community and critical-access hospital partnerships.

## Discussion

This study introduces the LRS as complementary infrastructure to the LHS, positioning systematic learning within and across research processes as a distinct domain of translational science [1,8,9]. By defining the LHS–LRS sociotechnical architecture (Figure 1), tracing the conceptual emergence of the LRS (Table 1), differentiating LHS and LRS functions across the NCATS Translational Science Spectrum (Table 2), and assessing institutional maturity through a formative institutional case (Table 3), we clarify how research workflows, governance, and data infrastructure can enable cross-study learning [7]. Together, these elements position the LRS as translational science infrastructure focused on research-process learning and provide a basis for future empirical evaluation.

The LRS develops from LHS scholarship by extending the focus of learning from care improvement to research process optimization at the portfolio-wide scale [17,25]. This distinction is analogous to the one between translational research and translational science, where the former defines research as the engine of health outcome improvement, and the latter defines the study of generalizable principles and operational capabilities that increase the efficiency and continuity of that engine [7]. When implemented effectively, translational science outputs accelerate translational research advances and, by extension, improve care.

CRMs represent one potential approach to assessing LRS maturity at the portfolio level. CRMs can summarize research-system performance across studies by aggregating operational indicators and outcome milestones, such as study activation time, cohort identification and recruitment efficiency, asset reuse, data-quality defect rates, analytic reproducibility, translation-to-care actions, and dissemination timelines. This approach is consistent with performance measurement frameworks that link strategic goals to measurable outcomes [29]. Implemented through an LRS dashboard, such measures could support continuous, scalable cross-study monitoring and operational feedback while highlighting the need for standards, risk adjustment, and multisite validation using objective operational data.

Persistent discontinuities across the translational spectrum remain a central challenge for clinical and translational science [2,10]. These discontinuities are multifactorial and may reflect limitations in preclinical models, insufficient rigor or reproducibility, incomplete reporting, regulatory or funding discontinuities, limited stakeholder engagement, and difficulty integrating research into clinical and operational workflows [11]. The LRS does not solve all sources of translational failure. Rather, it provides a mechanism for making selected research processes more measurable, reusable, governable, and feedback-enabled. In this narrower sense, LRS capabilities may help mitigate structural barriers that contribute to early-stage translational gaps, including but not limited to the T1 “valley of death” [30].

This work also underscores the absence of a widely adopted EHR-like operating environment for research [16]. The issue is not simply the absence of a single software platform. Rather, research data, metadata, regulatory workflows, protocols, analytic code, recruitment processes, biospecimens, imaging resources, staffing information, and study-performance measures often remain distributed across IRB systems, CTMS platforms, REDCap, EHR query tools, data warehouses, imaging repositories, biobanks, eRegulatory systems, analytics platforms, project-management tools, and compliance workflows [1,19]. This fragmentation limits cross-study measurement, asset reuse, portfolio-level monitoring, and rapid feedback.

The LRS framework addresses this barrier by specifying the capabilities needed for a more coherent research operating environment: shared measures and data standards, reusable assets and workflows, accountable governance, analytic monitoring, and mechanisms that connect research activity to implementation and practice. A mature LRS does not require a single electronic research record. However, an electronic research record or equivalent integrated architecture could accelerate LRS development by linking protocols, study metadata, data dictionaries, code, ontologies, access permissions, recruitment workflows, regulatory status, staffing, study performance, and analytic outputs.

### Limitations

This study has several limitations. The literature review was intended to synthesize representative works and clarify the conceptual lineage and candidate maturity indicators of the LRS rather than provide an exhaustive review. The formative use case reflects a single academic medical center with well-developed translational infrastructure, which limits generalizability to institutions with different resources, governance models, clinical partnerships, and community contexts. Although the analysis is grounded in one institution, it is situated within a broader sociotechnical ecosystem that includes CTRs and CTSAs, NIGMS, NCATS, the National Academy of Medicine, government and industry partners, patient advocacy organizations, and a wide range of research, clinical, institutional, and community collaborators. This context supports the relevance of the case beyond the local setting but does not eliminate concerns about external validity.

Several authors are embedded within the institution studied, introducing the potential for insider assumptions, selection bias, confirmation bias, and overinterpretation of local experience. In addition, milestone identification relied on institutional documentation supplemented by informant input, which may be subject to selection and reporting bias. Capability maturity was inferred primarily from infrastructure, governance, and process indicators rather than formal comparative evaluation or validated outcome measures.

Together, these limitations highlight the need for empirical validation of composite research metrics and multisite studies to assess scalability, reproducibility, equity, and impact across diverse institutional and network contexts [12,23].

### Conclusion

This work advances the conceptualization of the LRS as translational science infrastructure that enables systematic learning within and across research processes. The definition, conceptual framing, and capabilities presented here provide a structured approach for describing and assessing research-process learning. Although further development and empirical validation of CRMs are needed, our findings suggest that coordinated data infrastructure, governance alignment, and feedback-enabled workflows can support cross-study learning, reduce fragmentation across translational stages, and improve research efficiency. Future multi-institutional studies of CRMs and LRS implementation will be essential to determine whether the framework is scalable, reproducible, equitable, and associated with improved translational performance.

## Figure 1. Long description

Two, doubled circles intersecting at learning. The LRS inner circle contains data, evidence, and research practice, while the LHS inner circle contains data, evidence, and clinical practice. The LRS outer circle states that process improvement accelerates learning and contains translational science, while the LHS outer circle states that learning improves practice and contains translational research.

Navigate back to Figure 1..

## Table 1. Long description

A table summarizing seminal literature supporting the emergence of the Learning Research System (LRS) concept from Learning Health System (LHS) scholarship. The table has two columns: Citation and Core contribution. It contains 21 rows, each representing a different study or piece of literature. The Citation column lists the authors, year, and sometimes a reference number. The Core contribution column describes the main findings or contributions of each cited work. The table illustrates the progression from care-centered learning models to intermediate research-support infrastructures, and finally to enterprise-level, continuous learning. Early literature emphasized rapid-cycle clinical improvement and integrating research with routine care, with limited attention to cross-study process learning. Subsequent scholarship focused on embedded research within LHSs, expanding to practical applications for research process improvement through recruitment monitoring, risk-based oversight, and multi-institutional coordination. Contemporary scholarship treats research itself as an object of systematic learning, advancing cross-study measurement, asset reuse, structured feedback, and coordinated regulation consistent with LRS criteria across the translational spectrum. The ordering from top to bottom reflects increasing functional integration rather than strict linear stages.

Navigate back to Table 1..

## Table 2. Long description

A table with five rows and three columns comparing primary functions of Learning Health Systems (LHS) and Learning Research Systems (LRS) across different stages of research. The columns are labeled Stage, Primary LHS functions, and Primary LRS functions. The stages include T0: Basic and Foundational Research, T1: Preclinical Research, T2: Clinical Research, T3: Clinical Implementation, and T4: Public Health. Row 1: Stage, T0: Basic and Foundational Research; Primary LHS functions, Identification of unmet clinical needs from real-world evidence and practice; Primary LRS functions, Governance and regulatory compliance streamlining, protocol and workflow template development, data quality and analyses monitoring, educational needs and resource assessments. Row 2: Stage, T1: Preclinical Research; Primary LHS functions, Signal detection from clinical data; workflow-linked feasibility and pilot studies; Primary LRS functions, Streamlined cohort identification, testing via animal models, recruitment monitoring, feasibility study metrics, and adaptive protocol support. Row 3: Stage, T2: Clinical Research; Primary LHS functions, Pragmatic trials embedded in care; decision support implementation; real-world validation of interventions; Primary LRS functions, Trial execution monitoring, harmonization across sites using standard data models, and analytic reproducibility. Row 4: Stage, T3: Clinical Implementation; Primary LHS functions, Continuous learning cycles; comparative effectiveness research; operational optimization and quality improvement; Primary LRS functions, Post-market evidence generation; rapid linkage of research outputs to operational data; evaluation of adoption and implementation fidelity. Row 5: Stage, T4: Public Health; Primary LHS functions, Population outcomes monitoring; predictive analytics for risk stratification; disparity identification and equitable implementation at scale; Primary LRS functions, Linkage with public health, environmental, and community datasets; federated (privacy-preserving) analytics; community-engaged research.

Navigate back to Table 2..

## Table 3. Long description

This table details the chronological progression of Learning Health System maturity milestones from 2004 to 2026. It tracks institutional developments, moving from early governance and biobank creation to regional network expansion and enterprise data modernization. Each milestone is mapped across six key categories: primary capabilities, research-system functions, quantitative metrics, maturity contributions, and current operational gaps requiring ongoing feedback and refinement.

Navigate back to Table 3..
